# Huashi Runzao decoction for primary Sjögren disease: a double-blind, randomized controlled trial combined with m6A and m5C RNA modification analysis

**DOI:** 10.3389/fphar.2025.1618649

**Published:** 2025-10-31

**Authors:** Ziwei Huang, Tiantian Deng, Xiya Zhang, Chunxin Lei, Yan Zhang, Jianying Yang, Qian He, Jiahe Liao, Jiaqi Chen, Liwen Yang, Xiaofeng Gu, Jing Luo, Cheng Xiao, Qingwen Tao

**Affiliations:** ^1^ Traditional Chinese Medicine Department of Rheumatism, China-Japan Friendship Hospital, Beijing, China; ^2^ Qingdao Municipal Hospital, Qingdao, China; ^3^ Graduate School, Beijing University of Chinese Medicine, Beijing, China; ^4^ Biotechnology Research Institute, Chinese Academy of Agricultural Sciences, Beijing, China; ^5^ Institute of Clinical Medical Sciences, China-Japan Friendship Hospital, Beijing, China

**Keywords:** Sjögren disease, Huashi Runzao decoction, Chinese medicine, RNA methylation, randomized controlled trial

## Abstract

**Background:**

Huashi Runzao decoction (HRD), a Chinese herbal formula, has been used in clinical practice for patients with primary Sjögren disease (pSD) for years. However, the benefits of HRD for pSD have not been evaluated, and HRD epigenetic mechanism of action remains unexplored.

**Objectives:**

We conducted a double-blind, randomized, placebo-controlled clinical trial to evaluate the efficacy and safety of HRD in patients with pSD and to explore its epigenetic mechanism of action.

**Methods:**

The clinical scores (including oral dryness, dry eye, dryness, fatigue, and limb pain visual analogue scales scores and the ESSPRI) of pSD patients were recorded at baseline and every 4 weeks thereafter. The disease activity scores (including the ESSDAI and ClinESSDAI), exocrine gland function variables (including the result of Schirmer’s test and salivary flow rate), serological indices (ESR, CRP, IgG, IgA, and IgM) and short-form-36 health survey (SF-36) score were evaluated at baseline and 12 weeks later. Peripheral blood samples were collected from patients and healthy volunteers to determine RNA methylation (m6A and m5C) levels and analyse regulatory factor expression.

**Results:**

HRD improved exocrine gland function in pSD patients and increased saliva (*P* = 0.049) and tear (*P* = 0.005) secretion. It also improved patients’ perceptions of subjective symptoms, including oral dryness (*P* < 0.001), dry eye (*P* = 0.004), dryness (*P* = 0.001), and limb pain (*P* = 0.008) and yielded greater ESSPRIs (*P* = 0.001), reduced patients’ disease activity according to the ClinESSDAI (*P* = 0.038) and improved their quality of life. Moreover, HRD increased m6A levels and decreased m5C levels in pSD patients, and HNRNPA2B1 was identified as a potential key epigenetic regulator.

**Conclusion:**

HRD, a Chinese herbal medicine, may be a promising treatment for pSD, especially for glandular damage. The therapeutic effects of this decoction may be achieved by alteration of the HNRNPA2B1 gene, altering m6A and m5C levels in pSD patients.

## Introduction

Primary Sjögren disease (pSD) is a systemic autoimmune disease with a prevalence ranging from 0.3% to 0.8% in the Chinese population (Longhino et al., 2023; Xu et al., 2020), while a global meta-analysis revealed a total prevalence of 60.82 per 100,000 people (Qin et al., 2015). Patients with pSD often have a reduced functionality of exocrine glands such as salivary glands and lacrimal glands, with the pathological features of substantial lymphocytic infiltration in the glandular stroma, destruction of the glandular ducts, and atrophy (Nocturne and Mariette, 2013). The clinical manifestations of Sjögren disease (SjD) primarily include dry mouth and eyes, while some patients with severe SjD may experience multisystem involvement, including thrombocytopenia and interstitial lung disease, leading to a poor outcome (La Rocca et al., 2023; Luciano et al., 2024). Owing to its unclear aetiology and pathogenesis, however, there is currently a lack of definitive and effective therapeutic agents for pSD, and so the development of better treatment strategies is warranted. Through a multifaceted and multitarget approach and relatively minor side effects, Chinese medicine offers a treatment option for pSD that balances efficacy and safety (Liu H et al., 2023; Xu and Song, 2024). The herbal formula Huashi Runzao decoction (HRD), consisting of six different Chinese herbs, is derived from the combination of two traditional Chinese herbal formulas named Baihe Dihuang decoction and Baohe pill, with modifications and adaptations. HRD has been granted a Chinese national invention patent and has been used to relatively effectively treat patients with pSD in clinical practice for years, it can effectively improve the dry symptoms of patients with pSD and reduce disease activity (Wu et al., 2023). However, high-quality randomized controlled trials (RCTs) assessing the benefits of HRD for patients with pSD and explorations of the mechanisms underlying its clinical value are lacking.

N6-methyladenosine (m6A) and 5-methylcytosine (m5C) RNA modifications are the most prevalent types of mRNA transcription modification and play significant roles in various autoimmune diseases, such as rheumatoid arthritis and systemic lupus erythematosus (Cui et al., 2022; Dong et al., 2022). Recent studies have shown that the presence of the m6A modification in the peripheral blood mononuclear cells (PBMCs) of patients with pSD is significantly related to the pathogenesis of the disease (Thorlacius et al., 2023), suggesting that the regulators of the m6A and m5C modifications are extensively involved in mediating immune cell balance and the type I IFN signalling pathway (Xiao et al., 2022). For example, the level of METTL3, an m6A methyltransferase, is positively correlated with the degree of inflammation and disease activity in pSD patients, making it a potential biomarker of the disease (Cheng et al., 2022).

This study aimed to evaluate the therapeutic effects and safety of HRD in patients with pSD by a double-blind, randomized controlled clinical trial. Moreover, we explored the epigenetic mechanisms of action of HRD to broaden clinical treatment options for patients with pSD.

## Methods

### Trial overview

This was a single-centre, randomized, double-blind, placebo-controlled clinical trial. Participants were recruited from the outpatient and ward of China-Japan Friendship Hospital from 13 February 2023, to 13 March 2024. All the subjects who participated in this study voluntarily signed informed consent forms. This study was approved by the Ethics Committee of China-Japan Friendship Hospital (2022-KY-74-1) and is registered in the Chinese Clinical Trial Registry (ChiCTR2200061608). We confirm that our clinical trial follows the CONSORT standards (http://www.consort-state ment.org/) and *The Reporting Standard for Clinical Randomized Controlled Trials of Chinese Medicine Compounds* (2017 edition) (Cheng et al., 2017).

### Participants

Patients were recruited from the China-Japan Friendship Hospital. The inclusion criteria were as follows: (1) age 18–75 years; (2) fulfilling the 2016 American College of Rheumatology/European League Against Rheumatism (ACR/EULAR) classification criteria for primary Sjögren’s syndrome (Shiboski et al., 2017); (3) with positive auto-antibodies (antinuclear antibodies (ANA) or anti-Sjögren’s-syndrome-related antigen A (SSA) antibodies or anti-Sjögren’s-syndrome-related antigen B (SSB) antibodies or rheumatoid factor (RF)) and/or high immunoglobulin levels or accelerated erythrocyte sedimentation rates (ESR); (4) compliance with the traditional Chinese medicine syndrome of dampness and dryness; (5) informed and voluntarily signing of the informed consent form. The exclusion criteria were as follows: (1) diagnosis with other connective tissue diseases; (2) using glucocorticoids or biological immunosuppressive agents within the previous 3 months before recruitment; (3) pregnancy, planning for pregnancy or lactation; (4) severe mental illnesses or neurological disorders; (5) severe lesions in heart, brain, lungs, liver, kidneys, or haematopoietic system; (6) visual field defects, fundus lesions, and severe arrhythmias (such as atrial fibrillation, atrioventricular block); and (7) participating in other drug trials. In addition, all participants who completed the informed consent form and were screened for eligibility to enter the RCT were marked as dropouts, regardless of the time or reason for withdrawal, as long as they had not completed the treatment observation period specified in the protocol.

### Sample size

We adopted a superiority testing design method, and used PASS 15.0.5 software for sample size estimation. The HRD group and placebo group were set at a 1:1 ratio. The EULAR Sjögren’s Syndrome Patient-Reported Index (ESSPRI) was used as the primary efficacy endpoint. Previous clinical trial have shown that after intervention with HRD, the mean and standard deviation of ESSPRI scores in pSD patients were 2.53 and 1.20, respectively, and in the placebo group were 0.83 and 1.37, with α = 0.05, β = 0.1, SM = 1, and considering a 20% dropout rate, it was determined that 59 patients needed to be enrolled in each group, resulting in a total sample size of 118 patients.

### Intervention

The participants were randomly assigned to the HRD group or the placebo group at a 1:1 ratio. Participants in the HRD group received HRD and hydroxychloroquine sulfate, whereas those in the placebo group received HRD placebo and hydroxychloroquine sulfate. The participants in both the HRD and placebo groups were each administered one packet of the corresponding substance 30 min after breakfast and one packet each after dinner, whereas 200 mg of hydroxychloroquine sulfate was taken every 30 min after breakfast and 30 min after dinner. The raw materials for HRD were manufactured by Beijing Meikangtang Pharmaceutical Technology Co., Ltd., and their specific compositions are shown in Table 1. The HRD placebo was composed of 10% HRD, a flavouring agent, and an edible pigment. HRD and its placebo were prepared as Chinese herbal decoctions (100 mL/packet) by the Pharmacy Department of China-Japan Friendship Hospital. The hydroxychloroquine sulfate tablets were manufactured by Shanghai Shangyao Zhongxi Pharmaceutical Co., Ltd., and contained 100 mg of active ingredient per tablet (batch No. 119990264).

**TABLE 1:** Constituent medicinal materials in HRD.

No.	Chinese names	Latin names	Dose (g)
1	Di Huang	*Rehmannia glutinosa* Libosch	15
2	Bai He	*Lilium lancifiolium* Thunb	10
3	Fu Ling	*Poria cocos* (Schw.) Wolf	15
4	Zi Su Geng	*Perilla frutescens* (L.) Britt	10
5	Mai Ya	*Hordeum vulgare* L	10
6	Sang Ye	*Morus alba* L	5

The HRD was constructed as follows: (1) *Rehmannia glutinosa* Libosch (15 g), *Lilium lancifiolium* Thunb (10 g), *Poria cocos* (Schw.) Wolf (15 g), *Perilla frutescens* (L.) Britt (10 g), *Hordeum vulgare* L. (10 g) and *Morus alba* L. (5 g) herbs were mixed and soaked in cold water for 15 min (2) After boiling for 20 min, 200 mL of liquid was poured out from the filter residue. (3) Cold water was added to immerse the medicinal residue again, and the herbs were boiled for 20 min. Two hundred millilitres of liquid was then poured from the filtered residue. (4) The two liquids are mixed and divided evenly into two portions, morning and evening, to be taken warm after meals.

The preparation process for the HRD placebo was as follows: (1) A 0.02‰ bittering agent (sucrose octaacetate), 1.26‰ colouring agent (caramel pigment), 5.2‰ polyglucose, and 0.1‰ citric acid were added to a container, water was added, and the mixture was stirred evenly. (2) A total of 10% raw HRD material solution was added and mixed well to create a simulated substance that was similar in colour, odour, and appearance to HRD. (3) The substance was mixed thoroughly and boiled before distribution and packaging.

Upon enrolment, the participants underwent a comprehensive collection of demographic data and physical examinations. Subsequently, symptom and disease activity assessments and exocrine gland tests were performed, along with laboratory tests, including complete blood cell counts, hepatic and renal function tests, tests for assessing markers of immune inflammation, and electrocardiography.

All participants performed self-assessments of their symptoms every 4 weeks and kept a diary of their self-ratings. At the 12th week, they returned to the hospital for a comprehensive evaluation. Adverse reactions were continuously monitored and documented throughout the medication period. At the 24th week, follow-up was still continued to assess their symptom scores.

### Randomization and blinding

The participants were randomized by experts in research methodology from the Clinical Research Data and Project Management Platform at China-Japan Friendship Hospital using SAS 9.4 statistical software through a simple random method. Each participant was assigned a random number, which was kept in a sealed envelope by a designated individual not involved in the clinical trial of the research team. The participants, physicians and statisticians were unable to access any information that could disclose the group allocation of the participants through any means. When a participant was enrolled, he or she was contacted to provide them with the random number.

### Outcome measures

The primary outcome measure was the assessment of the salivary flow rate.

The secondary outcome measures included clinical scale indicators, obtained through visual analogue scales (VAS) (including the oral dryness VAS score, dry eye VAS score, fatigue VAS score, and limb pain VAS score), at all time points (the 4th, 8th, 12th, and 24th week). The overall symptom assessment was evaluated using the ESSPRI at all time points. Additionally, disease activity was assessed using the EULAR Sjögren’s Syndrome Disease Activity Index (ESSDAI) and the clinical ESSDAI (ClinESSDAI). Exocrine gland function was assessed with Schirmer’s test. In addition, immune-inflammatory indicators such as ESR, C-reactive protein (CRP) level, immunoglobulin G (IgG) level, IgA level and IgM level were measured. Quality of life was assessed using the short form-36 health survey (SF-36) and Sjögren’s tool for assessing response (STAR). Unless indicated otherwise, the above indicators were evaluated only at week 12.

### Sample collection

Peripheral blood samples were collected from included patients who gave additional informed consent before and after intervention. Meanwhile, samples were also collected from healthy volunteers with matched gender and age. All healthy volunteers were recruited from the Health Examination Center of China-Japan Friendship Hospital, and their samples were only collected at the time of enrollment. Leukocytes from the peripheral blood were isolated using a density gradient centrifugation, followed by RNA extraction from the isolated leukocytes with TRIzol reagent.

### m6A and m5C RNA modification and regulator analysis

The RNA concentration and purity of samples were measured with a NanoDrop One ultramicro spectrophotometer (Thermo Fisher Scientific, Waltham, MA, United States). The experiments and data analysis for m6A and m5C RNA modifications were conducted by Aksomics through their liquid chromatography–mass spectrometry (LC-MS) mRNA modification detection service. Five postintervention samples each from the placebo-treated and HRD-treated patients, as well as five from healthy volunteers, were selected from the samples for RNA modification regulator analysis. The experimental procedures and data analysis were conducted by Aksomics using a Human Epitranscriptomics PCR array.

### Epitranscriptomic analysis

The t-test was conducted to assess the impact of HRD on the m6A and m5C RNA modifications in pSD patients. Differential expression gene (DEG) analysis was carried out according to a screening criterion of |logFC| >1, adjusted *P* < 0.05, to identify key regulators in the experiment. The target genes of these key regulators were subsequently searched in the RM2Target database (http://rm2target.canceromics.org), and validated target genes were selected for GO enrichment analysis. Finally, Spearman analysis was employed to evaluate the associations between key regulators and patient clinical characteristics.

### Statistical tests

GraphPad Prism eight was used for statistical analysis. Count data are described as [*n* (%)]. Normally distributed measurement data are described as ( $\bar{x}\pm s$ ), whereas data not conforming to a normal distribution are described as [*M* (*P*25, *P*75)]. Intergroup comparisons of count data were performed with the chi-square test, Yates’ corrected chi-square test, or Fisher’s exact test, as appropriate. If the data could be described with a parametric model, the two-sample independent t-test was employed for between-group comparisons; otherwise, the Mann‒Whitney U test was used. For within-group sample data that could be described with a parametric model, the paired t-test (two-tailed) was used for analysis; if not, the Wilcoxon matched-pairs signed-rank test was employed. Repeated measures indicators were evaluated with a mixed-effect model for repeated measures (MMRM) using the “nlme” R software package. A two-sided *P* value <0.05 was considered to indicate statistical significance.

## Results

### Patient characteristics

**FIGURE 1:**
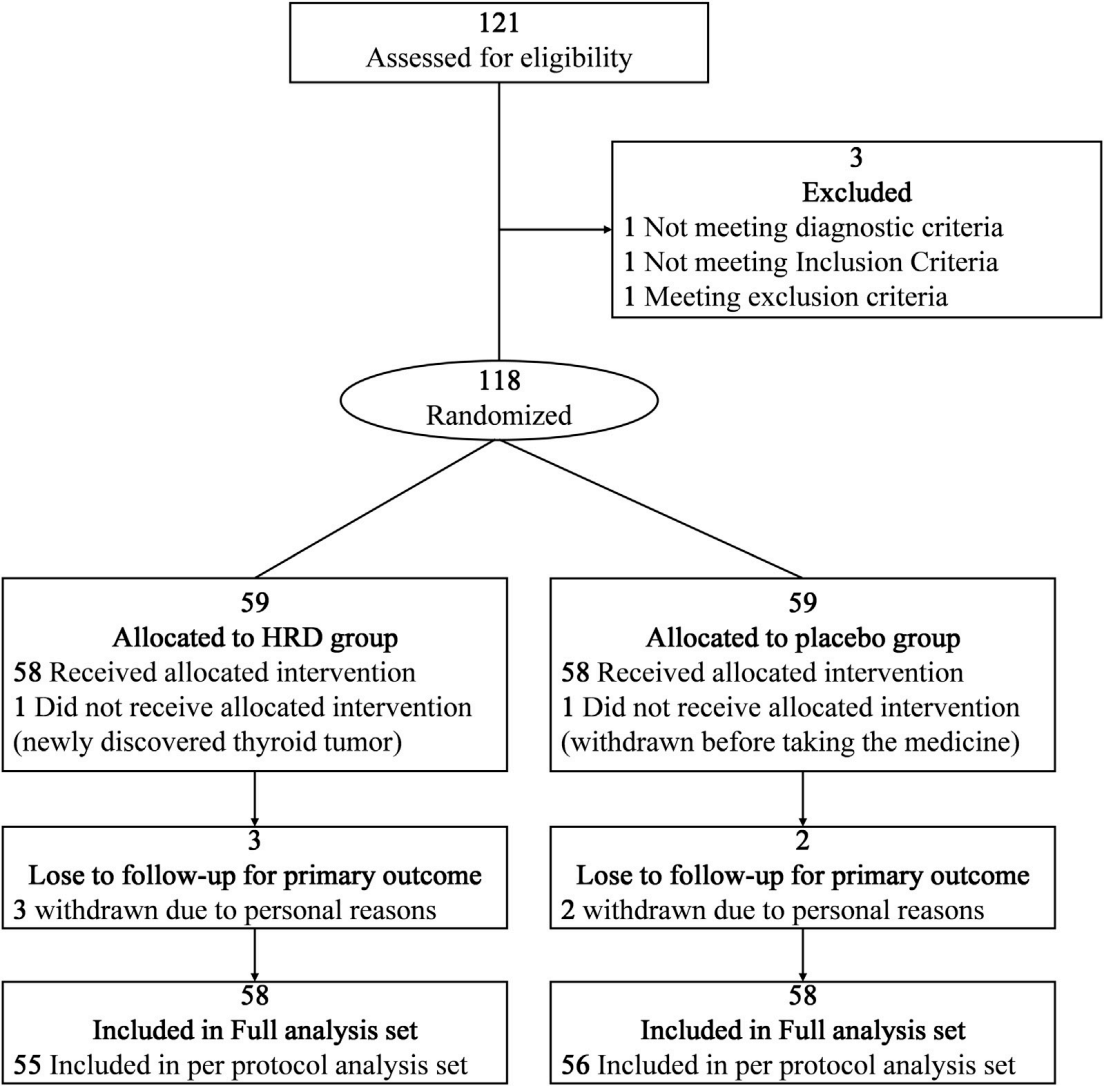
Flow diagram of the trial design.

From 13 February 2023, to 13 March 2024, a total of 121 potential participants at the China-Japan Friendship Hospital were assessed for eligibility, among whom 118 participants entered the RCT and were assigned to the HRD group or placebo group at a 1:1 ratio (Figure 1). In the HRD group, one patient withdrew from the trial before taking the medicine because a thyroid tumor was discovered simultaneously. In placebo group, one patient withdrew from the trial before taking the medicine due to personal reason. Therefore, 116 patients were included in this study. However, three patients in HRD group and two patients in placebo group withdrew from the trial after taking the medicine due to personal reasons. There were 111 patients included in per protocol analysis set. No adverse reaction symptoms occurred during the medication period of the above five patients who have withdrawn from the trial. All the 116 patients completed the assessment of symptom scores at the 24th week. There were no statistically significant differences in the baseline levels of each outcome indicator between the two groups (Table 2).

**TABLE 2:** Baseline characteristics of the patients.

Baseline characteristic	Patients, no. (%)			*P* value
	HRD (*n* = 58)	Placebo (*n* = 58)	Total (*n* = 116)	
Gender				0.361
Male	1 (1.7)	4 (6.9)	5 (4.3)	
Female	57 (98.3)	54 (93.1)	111 (95.7)	
Age, mean ± SD, year	53.7 ± 13.5	55.2 ± 9.7	54.5 ± 11.7	0.881
Age of onset, mean ± SD, year	47.5 ± 12.5	48.4 ± 10.0	48.0 ± 11.3	0.864
Course of disease, median (range), month	48 (14.75–99)	60 (28–120)	60 (24–115.5)	0.235
Race				0.166
Han	53 (91.4)	48 (82.8)	101 (87.1)	
Other^a^	5 (8.6)	10 (17.2)	15 (12.9)	
BMI, mean ± SD	22.3 ± 3.7	23.4 ± 3.4	22.9 ± 3.6	0.070
Medication history				
Hydroxychloroquine sulfate	27 (46.6)	25 (43.1)	52 (44.8)	0.709
Iguratimod	5 (8.6)	2 (3.4)	7 (6.0)	0.435
Tripterygium Polyglycosid	0	3 (5.2)	3 (2.6)	0.242
Paeony Capsule	5 (8.6)	4 (6.9)	9 (7.8)	1.000
Chinese herbal decoction	5 (8.6)	2 (3.4)	7 (6.0)	0.435
Other^b^	0	2 (3.4)	2 (1.7)	0.496
Smoking history	3 (5.2)	1 (1.7)	4 (3.4)	0.611
Drinking history	1 (1.7)	2 (3.4)	3 (2.6)	1.000
Family history of pSD	4 (6.9)	5 (8.6)	9 (7.8)	1.000

Gender, race, medication history, smoking history, drinking history and family history were expressed as ( $\bar{x}\pm s$ ), and were statistically analyzed by chi-square test; Age, age of onset, BMI were expressed as [*n* (%)], and were statistically analyzed by the two-sample independent t-test; Course of disease was expressed as [*M* (*P25*, *P75*)], and was statistically analyzed by Mann‒Whitney U test; HRD, huashi runzao decoction; BMI, body-mass index; pSD, primary Sjögren disease.

^a^ Other race included minority nationality.

^b^ Other medication history included glucocorticoids and cyclophosphamide.

### Primary outcome

**FIGURE 2:**
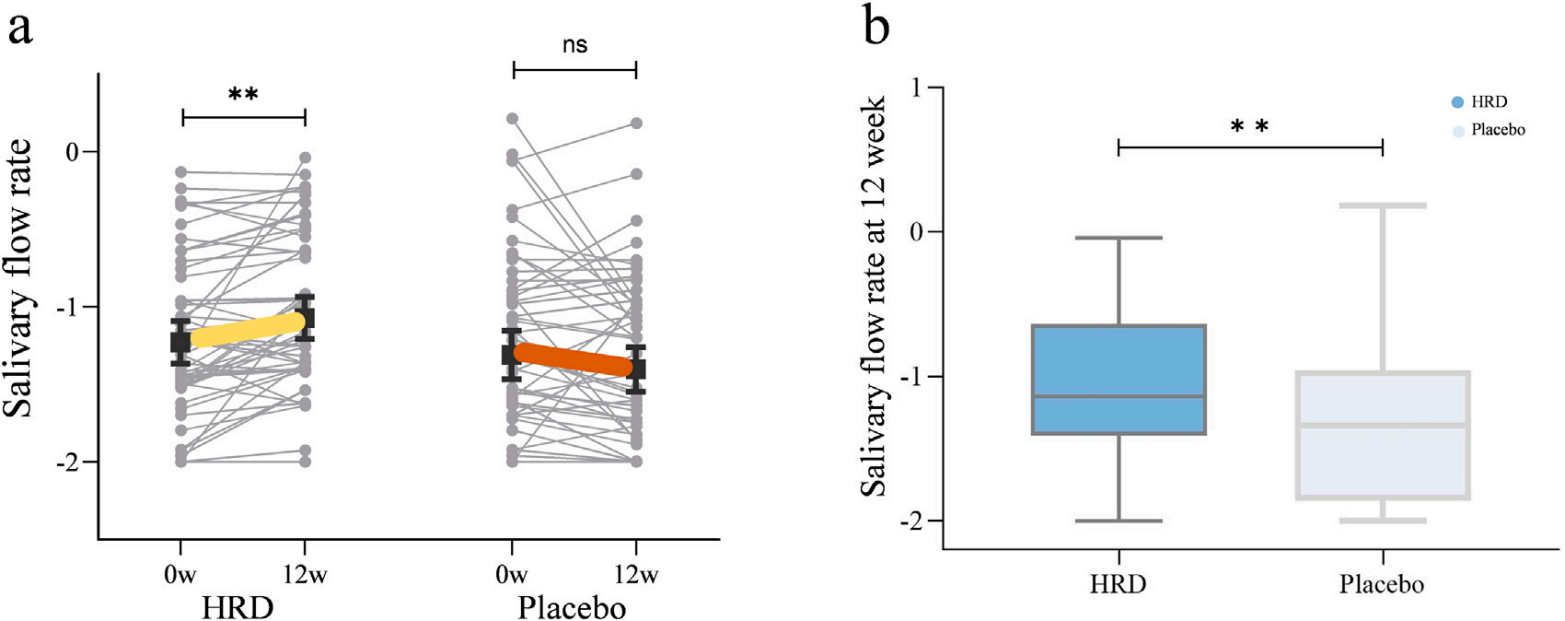
The influence of HRD on salivary flow rate. HRD, Huashi Runzao decoction; **(a)** Data were analyzed by Wilcoxon matched-pairs signed-rank test, paired half-violin plot showing the increase of salivary flow rate in the HRD group after 12 weeks of treatment (*P* < 0.01). **(b)** Data were analyzed by Mann‒Whitney U test, boxplot showing a more significant increase of salivary flow rate in the HRD group compared to the placebo group at the 12th week (*P* < 0.01).

Table 3 lists all outcome results for the HRD group and placebo group. After 12 weeks of HRD treatment, the salivary flow rate was greater than at baseline (*P* = 0.002), whereas no differences from baseline were observed in the placebo group (*P* = 0.678). The salivary flow rate from week 0 to week 12 in the HRD group (median improvement: 0.009) significantly differed from that in the placebo group (median improvement: 0.000), *P* = 0.049 (Figure 2).

**TABLE 3:** Clinical and laboratory indicators of the HRD group and placebo group at each visit.

Measures		HRD (*n* = 58)					Placebo (*n* = 58)				
		0 w	4 w	8 w	12 w	24 w	0 w	4 w	8 w	12 w	24 w
Salivary flow rate, mL/min		0.04 (0.02, 0.11)	-	-	0.06 (0.03, 0.22)	-	0.03 (0.01, 0.11)	-	-	0.02 (0.00, 0.10)	-
Clinical scale indicators	Oral dryness VAS score	5.0 (4.0, 8.0)	4.0 (3.0, 5.3)	4.0 (2.8, 5.0)	4.0 (2.0, 5.0)	4.5 (2.0, 6.0)	5.0 (3.8, 7.0)	5.5 (4.0, 7.0)	5.5 (4.0, 7.0)	5.0 (4.0, 7.0)	5.0 (3.0, 6.0)
	Dry eye VAS score	6.0 (4.0, 7.0)	5.5 (3.0, 7.0)	5.0 (3.0, 7.0)	5.0 (3.0, 6.0)	5.0 (3.0, 6.0)	5.0 (3.0, 6.0)	5.0 (3.0, 6.0)	4.0 (3.0, 6.0)	5.0 (3.0, 6.0)	4.5 (3.0, 5.0)
	Dryness VAS score	6.0 (4.0, 7.0)	5.0 (4.0, 6.0)	5.0 (3.0, 6.0)	5.0 (3.0, 6.0)	5.0 (3.0, 6.0)	5.0 (3.8, 7.0)	5.0 (3.0, 6.3)	5.0 (3.0, 6.0)	5.0 (4.0, 6.0)	5.0 (3.8, 6.0)
	Fatigue VAS score	5.0 (3.0, 7.0)	5.0 (3.0, 7.0)	5.0 (3.0, 6.0)	4.0 (2.0, 6.0)	4.0 (2.0, 6.0)	5.0 (2.8, 6.0)	3.5 (2.0, 6.0)	4.0 (2.0, 5.3)	3.5 (2.0, 5.0)	4.0 (2.0, 5.0)
	Limb pain VAS score	3.0 (0.8, 5.0)	3.0 (1.0, 5.0)	2.0 (1.0, 5.0)	2.0 (0.0, 5.0)	2.5 (0.0, 4.0)	3.0 (0.0, 5.0)	3.0 (0.8, 5.0)	3.0 (0.0, 5.0)	3.5 (0.0, 5.0)	3.0 (1.0, 4.0)
ESSPRI		4.7 (3.3, 6.0)	4.3 (2.7, 5.8)	4.0 (2.7, 5.3)	3.7 (2.6, 5.0)	3.7 (2.7, 5.0)	4.0 (2.7, 5.4)	3.7 (2.7, 5.4)	3.7 (2.7, 5.0)	4.0 (2.7, 5.3)	3.8 (2.7, 5.0)
ESSDAI		3.0 (1.0, 5.0)	-	-	2.0 (1.0, 3.3)	-	3.0 (0.0, 5.0)	-	-	2.0 (1.0, 5.0)	-
ClinESSDAI		2.0 (0.0,4.0)	-	-	0.0 (0.0, 2.0)	-	2.0 (0.0, 6.0)	-	-	2.0 (0.0, 6.0)	-
Schirmer’s test	Left eye	3.5 (0.0, 6.0)	-	-	4.0 (0.0, 7.5)	-	5.0 (0.0,10.0)	-	-	3.5 (0.0, 9.3)	-
	Right eye	2.5 (0.0,4.0)	-	-	4.0 (0.0, 7.3)	-	3.0 (1.0,7.3)	-	-	4.0 (0.0, 8.0)	-
	Average	2.5 (0.9, 4.5)	-	-	3.5 (1.5, 7.5)	-	4.0 (1.0,0.8.4)	-	-	4.0 (0.0, 8.0)	-
ESR, mm/h		19.5 (8.0, 30.25)	-	-	17.0 (10.0, 27.3)	-	16.0 (10.8, 33.0)	-	-	16.0 (9.8, 30.0)	-
CRP, mg/l		1.8 (1.4, 2.6)	-	-	2.2 (1.5, 3.0)	-	0.2 (0.2, 0.3)	-	-	2.2 (1.5, 3.3)	-
IgG, mg/dL		17.5 (15.3, 30.0)	-	-	18.5 (14.7, 20.7)	-	16.4 (14.1, 20.5)	-	-	17.1 (13.1, 20.1)	-
IgA, mg/dL		3.2 (2.4, 3.9)	-	-	3.2 (2.3, 3.9)	-	2.9 (2.3, 3.8)	-	-	3.0 (2.2, 3.6)	-
IgM, mg/dL		1.1 (0.8, 1.7)	-	-	1.2 (0.9, 1.6)	-	1.1 (0.7, 1.6)	-	-	1.0 (0.7, 1.5)	-

All data were expressed as [*M* (*P25*, *P75*)], and were statistically analyzed by Mann‒Whitney U test; HRD, huashi runzao decoction; VAS, visual analogue scale; ESSPRI, European League Against Rheumatism Sjögren’s Syndrome Patient-Reported Index; ESSDAI, European League Against Rheumatism Sjögren’s Syndrome Disease Activity Index; ClinESSDAI, clinical European League Against Rheumatism Sjögren’s Syndrome Disease Activity Index; ESR, erythrocyte sedimentation rate; CRP, C-reactive protein; IgG, immunoglobulin G; IgA, immunoglobulin A; IgM, immunoglobulin M.

### Secondary outcome

**FIGURE 3:**
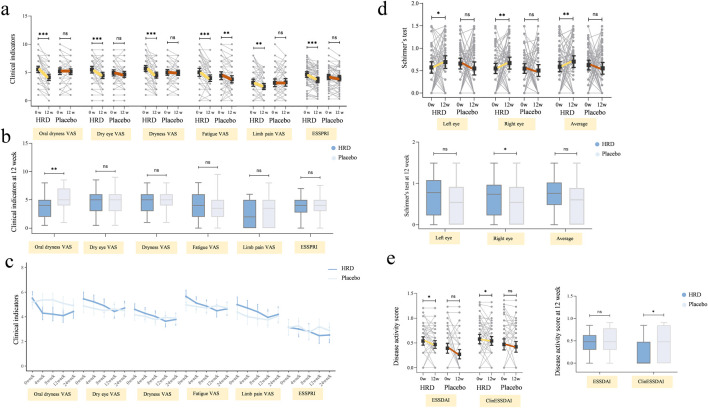
The influence of HRD on clinical indicators, tear gland function and disease activity. HRD, Huashi Runzao decoction; **(a)** Data were analyzed by Wilcoxon matched-pairs signed-rank test, paired half-violin plot showing the decrease in HRD after 12 weeks on oral dryness VAS (*P* < 0.001), dry eye VAS (*P* < 0.001), Dryness VAS (*P* < 0.001), Fatigue VAS (*P* < 0.001), Limb pain VAS (*P* < 0.01), and ESSPRI (*P* < 0.001). **(b)** Data were analyzed by Mann‒Whitney U test, boxplot showing there is a more significant decrease in oral dryness VAS between HRD and Placebo at the 12th week (*P* < 0.01), and no statistically significant difference in other clinical indicators (*P* > 0.05). **(c)** Data were analyzed by mixed-effect model, MMRM showing the fatigue VAS in both groups are gradually decreasing, with a consistent trend (*P* > 0.05). The other clinical indicators in HRD decreased significantly compared to Placebo (*P* < 0.001), with significant interaction effects at the time points 0w-12w (*P* < 0.05). **(d)** Data were analyzed by Wilcoxon matched-pairs signed-rank test, up-paired half-violin plot showing the decrease in the Schirmer’s test including left eye (*P* < 0.05), right eye (*P* < 0.01), and average (*P* < 0.01) in HRD after 12 weeks. Data were analyzed by Mann‒Whitney U test, down-boxplot showing a more significant decrease in right eye Schirmer’s test in HRD compared to Placebo at the 12th week (*P* < 0.05). **(e)** Data were analyzed by Wilcoxon matched-pairs signed-rank test, left-paired half-violin plot showing the decrease in ESSDAI and ClinESSDAI in HRD after 12 weeks (*P* < 0.05). Data were analyzed by Mann‒Whitney U test, right-boxplot showing a more significant decrease in ClinESSDAI in HRD compared to Placebo at the 12th week (*P* < 0.05).

In terms of secondary outcome measures, the clinical scale indicators in the HRD group, including the oral dryness VAS score (*P* < 0.001), dry eye VAS score (*P* < 0.001), dryness VAS score (*P* < 0.001), fatigue VAS score (*P* < 0.001), limb pain VAS score (*P* = 0.003) and ESSPRI score (*P* < 0.001), all decreased from baseline to week 12. After treatment, the HRD group showed significant improvements in the oral dryness (median improvement: 1.0 for the HRD group and 0.0 for the placebo group, *P* < 0.001), dry eye (median improvement: 1.0 for the HRD group and 0.0 for the placebo group, *P* = 0.004), dryness (median improvement: 1.0 for the HRD group and 0.0 for the placebo group, *P* = 0.001), and limb pain VAS scores (median improvement: 0.5 for the HRD group and 0.0 for the placebo group, *P* = 0.008), and the ESSRRI (median improvement: 1.0 for the HRD group and 0.0 for the placebo group, *P* = 0.001) relative to the placebo group, whereas there was no difference in the fatigue VAS score between the two groups (median improvement: 1.0 for the HRD group and 1.0 for the placebo group, *P* = 0.238) (Figures 3a,b). MMRM analysis revealed that the fatigue VAS scores of the HRD group and the placebo group gradually decreased over the treatment period but rebounded during the follow-up period with similar trends. Moreover, there was no group and time point interaction effect. However, during the treatment period, other clinical scale indicators in the HRD group gradually decreased over time. At the 12th week, the decrease in the HRD group was more significant than that in the placebo group, and significant group and time point interaction effects were observed over the entire trial period (Table 4; Figure 3c).

**TABLE 4:** Mixed-effects model for repeated measures (MMRM) of clinical symptom in two groups.

	Oral dryness VAS score	Dry eye VAS score	Dryness VAS score	Fatigue VAS score	Limb pain VAS score	ESSPRI
Group	−0.33 (0.39)	−0.57 (0.40)	−0.69 (0.36)	−0.60 (0.44)	−0.02 (0.45)	−0.44 (0.33)
0 w-4 w	−2.60 (0.34)^***^	−0.28 (0.37)	−0.95 (0.38)^*^	−0.17 (0.44)	−0.41 (0.54)	−0.51 (0.33)
0 w-8 w	−2.78 (0.34)^***^	−0.72 (0.37)	−1.33 (0.38)^***^	−0.67 (0.44)	−0.26 (0.54)	−0.75 (0.33)^*^
0 w-12 w	−2.74 (0.34)^***^	−1.78 (0.37)^***^	−2.28 (0.38)^***^	−1.43 (0.44)^**^	−1.41 (0.54)^**^	−1.70 (0.33)^***^
0 w-24 w	−1.88 (0.34)^***^	−1.10 (0.37)^**^	−1.78 (0.38)^***^	−1.28 (0.44)^**^	−1.00 (0.54)	−1.35 (0.33)^***^
Group × 0 w-4 w	1.40 (0.21)^***^	0.03 (0.23)	0.41 (0.24)	−0.10 (0.28)	0.28 (0.34)	0.19 (0.21)
Group × 0 w-8 w	1.48 (0.21)^***^	0.17 (0.23)	0.53 (0.24)^*^	0.09 (0.28)	−0.07 (0.34)	0.18 (0.21)
Group × 0 w-12 w	1.33 (0.21)^***^	0.76 (0.23)^**^	1.10 (0.24)^***^	0.40 (0.28)	0.74 (0.34)^*^	0.74 (0.21)^***^
Group × 0 w-24 w	0.80 (0.21)^***^	0.36 (0.23)	0.76 (0.24)^**^	0.48 (0.28)	0.38 (0.34)	0.54 (0.21)^**^

Value indicate the estimated effect (β) and corresponding standard error (SE); VAS, visual analogue scale; ESSPRI, European League Against Rheumatism Sjögren’s Syndrome Patient-Reported Index.

*indicates *P* < 0.05.

**indicates *P* < 0.01.

***indicates *P* < 0.001.

With respect to disease activity, glandular assessment, and serological parameters, after 12 weeks of HRD treatment, the ESSDAI (*P* = 0.022) and ClinESSDAI (*P* = 0.011) were lower than at values, whereas no such differences were observed in the placebo group (*P* = 0.094 and *P* = 0.112, respectively). There was no difference in the ESSDAI or the ClinESSDAI between the two groups at week 0, but there was a significant difference in the ClinESSDAI between the HRD group and the placebo group at week 12 (median values: 0.0 and 2.0, respectively; *P* = 0.038) (Figure 3e).

In terms of glandular function, in addition to the primary outcome measure of the salivary flow rate, there were also significant changes in the HRD group in the results of Schirmer’s test. Compared with those at week 0, the results for the HRD group were significantly greater at week 12, including those of the left eye (*P* = 0.017) and right eye (*P* = 0.001) and their mean values (*P* = 0.001), whereas those of the placebo group were not different from those at baseline. The change in the Schirmer’s test result from week 0 to week 12 in the HRD group (median improvement: 0.5) significantly differed from that of the placebo group (median improvement: 0.0), *P* = 0.005, both for the left eye (median improvement: 0.0 and 0.0, respectively, *P* = 0.015) and the right eye (median improvement: 1.0 and 0.0, respectively, *P* = 0.013) (Figure 3d).

None of the serological parameters were significantly different between the two groups before treatment (Supplementary Figure 1).

The scores for each dimension in the assessment of patients’ quality of life are shown in Table 5. The HRD group showed significant differences between week 0 and week 12 in four dimensions: emotional function (*P* = 0.048), physical pain (*P* = 0.026), overall health (*P* = 0.007), and health change (*P* < 0.001), whereas the placebo group only showed improvement in the health change dimension after 12 weeks of treatment compared with baseline (*P* = 0.001) (Supplementary Figure 2).

**TABLE 5:** Dimensions of SF-36 scale of the HRD group and placebo group before and after treatment.

Dimension	HRD (*n* = 58)		Placebo (*n* = 58)	
	0 w	12 w	0 w	12 w
Physical functioning	90.0 (75.0, 95.0)	90.0 (80.0, 95.0)	90.0 (76.3, 95.0)	90.0 (76.3, 95.0)
Role-physical	75.0 (25.0, 100.0)	100.0 (25.0, 100.0)	100.0 (25.0, 100.0)	100.0 (50.0, 100.0)
Bodily pain	77.8 (66.7, 88.9)	88.9 (66.7, 100.0)	77.8 (58.4, 88.9)	77.8 (66.7, 88.9)
General health	40.0 (30.0, 60.0)	50.0 (40.0, 65.0)	50.0 (35.0, 65.0)	52.5 (36.3, 70.0)
Vitality	65.0 (45.0, 75.0)	65.0 (50.0, 80.0)	70.0 (46.3, 80.0)	70.0 (55.0, 80.0)
Social functioning	75.0 (62.3, 87.5)	75.0 (62.5, 87.5)	75.0 (62.5, 100.0)	87.5 (62.5, 100.0)
Role-emotional	66.7 (0.0, 100.0)	66.7 (33.3, 100.0)	100.0 (33.3, 100.0)	100.0 (66.7, 100.0)
Mental health	72.0 (56.0, 84.0)	72.0 (60.0, 84.0)	74.0 (61.0, 84.0)	76.0 (56.0, 84.0)
Reported health transition	25.0 (25.0, 50.0)	50.0 (50.0, 75.0)	50.0 (25.0, 75.0)	75.0 (50.0, 75.0)

All data were expressed as [*M* (*P25*, *P75*)] and were statistically analyzed by Mann‒Whitney U test; HRD, huashi runzao decoction.

Among the 116 patients included in this study, three patients in HRD group and two patients in placebo group refused to undergo the examination and assessment at the 12th week due to personal reasons. Therefore, the STAR scale was used to evaluate a total of 111 participants in the two groups, with six individuals in the HRD group and three in the placebo group meeting the standard of clinical efficacy. No significant difference was observed between the groups.

In the study, a total of 11 participants experienced adverse events, with three occurring in the HRD group and eight in the placebo group. In the HRD group, two participants reported diarrhea and one reported nausea and vomiting. In the placebo group, three participants reported diarrhea, two reported nausea, and three reported rash. All adverse events resolved spontaneously and were considered unrelated to the intervention. There was no significant difference in the incidence of adverse event between the groups.

### Effect of HRD on the levels of m6A and m5C in pSD patients

**FIGURE 4:**
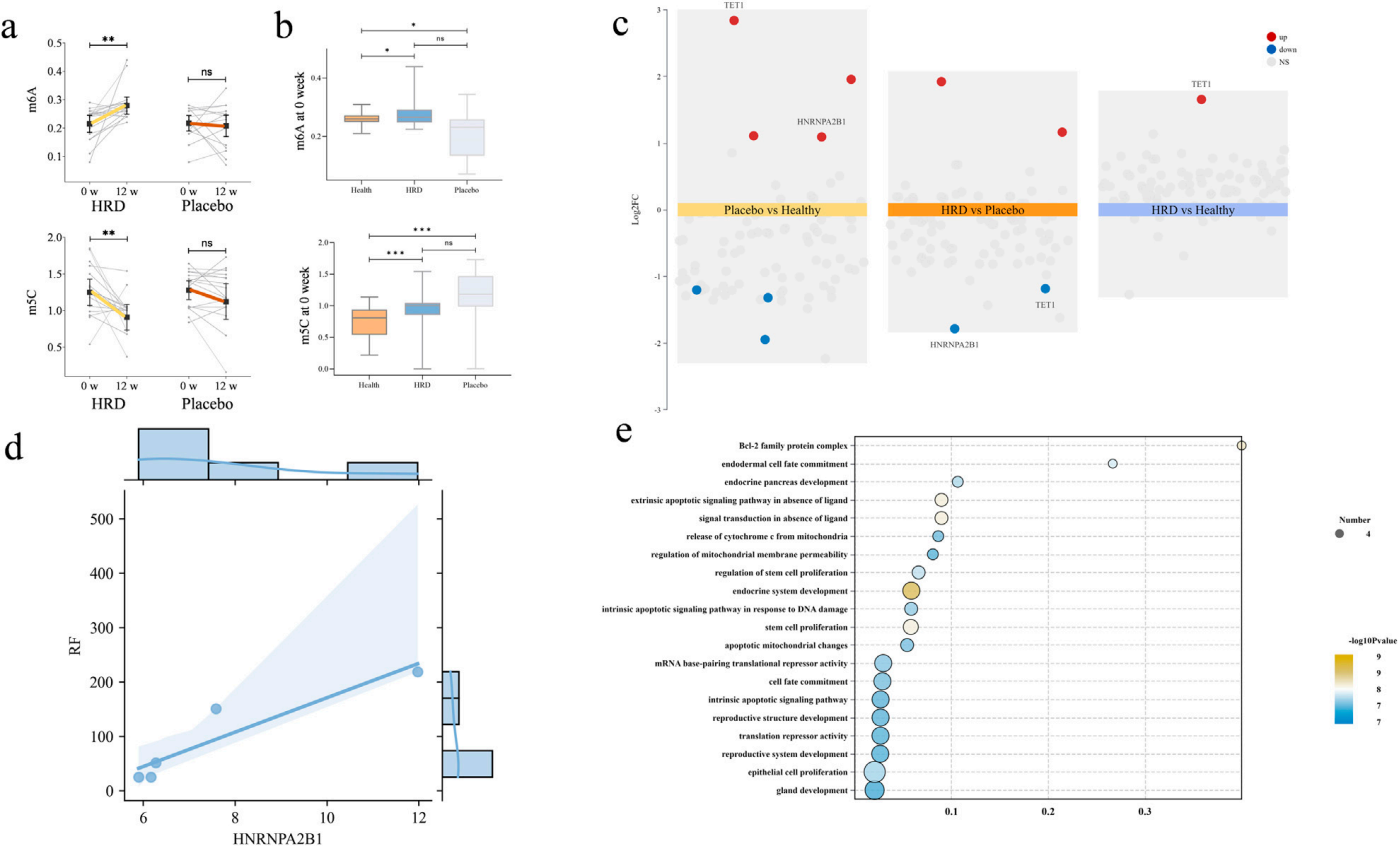
The influence of HRD on m6A, m5C and regulators. HRD, Huashi Runzao decoction; **(a)** All data were analyzed by Wilcoxon matched-pairs signed-rank test, up-paired half-violin plot showing the increase of m6A level in HRD after 12 weeks (*P* < 0.01), and down-paired half-violin plot showing the decrease of m5C level in HRD after 12 weeks; **(b)** All data were analyzed by Mann‒Whitney U test, up-boxplot showing at 0weeks, the levels of m6A in both HRD and Placebo groups were lower than those in HC, with no significant difference between the two groups and down-boxplot showing at 0weeks, the levels of m5A in both HRD and Placebo groups were higher than those in HC, with no significant difference between the two groups; **(c)** The differential regulators expression analysis revealed that after treatment, the expression of TET1 and HNRNPA2B1 were upregulated in Placebo compared to HC. In the HRD group, both factors were downregulated compared to the placebo group, while TET1 was upregulated compared to the control group; **(d)** Each point represents an individual patient (*n* = 5), and statistical analysis was performed using Spearman correlation (*r* = 0.975, *P* < 0.01); **(e)** Go analysis for target genes of HNRNPA2B1.

To further investigate the epigenetic mechanisms underlying the effects of HRD treatment in pSD, a total of 93 peripheral blood samples from patients and healthy volunteers who gave additional informed consent were collected in this study. After excluding patients who only provided blood samples once, 70 peripheral blood samples from patients before and after treatment (including 18 patients in HRD group and 17 patients in placebo group) and 18 peripheral blood samples from 18 healthy volunteers were collected. All of the 88 samples were used to examine the levels of m6A and m5C in the PBMCs. Compared with healthy volunteers, individuals in both the HRD group and the placebo group presented with lower m6A levels and higher m5C levels before treatment (*P* < 0.05). Compared with the corresponding levels at baseline, patients with pSD had increased m6A levels (*P* = 0.003) and decreased m5C levels (*P* = 0.003) after HRD treatment, whereas no significant differences were observed in the placebo group posttreatment (Table 6; Figures 4a,b).

**TABLE 6:** m6A and m5C expression levels of three groups before and after treatment.

Group	m6A level		m5C level	
	0 w	12 w	0 w	12 w
HRD group	0.24 (0.17, 0.25)	0.26 (0.25, 0.29)	1.27 (0.96, 1.55)	1.01 (0.81, 1.05)
Placebo group (SD)	0.22 (0.05)	0.21 (0.08)	1.29 (0.25)	0.92 (0.35)
Health volunteer (SD)	0.26 (0.02)	-	0.73 (0.26)	-

HRD, huashi runzao decoction; m6A, N6-methyladenosine; m5C, 5-methylcytosine.

### HRD may change methylation abnormalities by regulating HNRNPA2B1

We compared the differentially expressed regulators among healthy volunteers, the HRD group and the placebo group and found that HNRNPA2B1 and TET1 were significantly upregulated in the placebo group; moreover, both regulators were significantly downregulated after HRD treatment. Further comparison of the differentially expressed regulators between the HRD group and healthy subjects revealed that TET1 was still upregulated, but there was no significant difference in the expression of HNRNPA2B1 (Figure 4c). GO enrichment analysis of the target genes revealed that they were primarily enriched in “apoptosis”, which also included gland development and other features related to pSD (Figure 4d). Finally, Spearman correlation analysis revealed that the expression of HNRNPA2B1 was significantly correlated with the serum RF level in patients with HRD (*r* = 0.975, *P* < 0.01) (Figure 4e).

## Discussion

Most pSD patients produce autoantibodies *via* excessive activation of B cells, which also causes an increase in the levels of certain inflammatory factors (Zhan et al., 2023), leading to high disease activity, systemic disease manifestations and adverse outcomes (Arvidsson et al., 2024). Patients with high disease activity receive more frequent treatment with immunosuppressants, glucocorticoids, and biologics (Nguyen et al., 2024), but the degree of clinical symptom relief is limited (Assy et al., 2022; Chowdhury et al., 2021; Gottenberg et al., 2014). SjD patients mainly present with a series of clinical symptoms caused by involvement of exocrine glands, such as oral dryness and dry eye, which greatly reduce patients’ quality of life. Therefore, improving patient symptoms is one of the main goals in the development of SjD drugs (Assy et al., 2022; Chowdhury et al., 2021; Rihab et al., 2023). In recent years, RCT evaluations, including those involving the use of biologics, have shown that drug therapy for SjD exocrine gland involvement is often ineffective (Price et al., 2022). Chinese herbal decoctions, an element of traditional Chinese medicine—whose history spans thousands of years—can improve immune balance by regulating the body’s qi, blood, yin, and yang (Jiang et al., 2024; Liang et al., 2023). In clinical practice, traditional Chinese medicine is often used to treat SS, especially for improving the function of the exocrine glands (Wei et al., 2021). Moreover, a previous nonrandomized controlled clinical observation conducted by our team revealed that HRD was superior to hydroxychloroquine sulfate for improving clinical symptoms (Wu et al., 2023).

The results of clinical trials have shown that HRD combined with hydroxychloroquine sulfate can significantly improve the salivary flow rate, which objectively reflects the salivary gland function of the patient. The ACR/EULAR also suggests that the treatment response for dry mouth should focus on evaluating the salivary flow rate and rely less on patients’ subjective feelings, as these may be biased due to external environmental changes and personal factors (Ramos-Casals et al., 2020). In addition, improvements in dryness symptoms in the HRD group were also identified in the evaluation of lacrimal gland function. Schirmer’s test generally takes the average of the results from the left and right eyes as the test result. At week 12, the results of Schirmer’s test in the HRD group were significantly better than those before treatment and better than that in the placebo group. A systematic review and an RCT also revealed that traditional Chinese medicine demonstrates advantages in improving exocrine gland function (Chang et al., 2021; Liu Y et al., 2023).

The clinical symptoms of patients, such as oral dryness, dry eyes, dryness and limb pain, were alleviated to significantly better degrees than those of patients treated with hydroxychloroquine sulfate alone, which is consistent with the results of previous clinical trials (Gottenberg et al., 2014; Yoon et al., 2016). Moreover, our results showed that both HRD and the placebo improved patients’ fatigue symptoms with comparable effects. These findings demonstrate that HRD had no additional effect on lowering the fatigue VAS score and that improvements to this symptom were mainly due to the use of hydroxychloroquine sulfate. Previous studies have shown that hydroxychloroquine sulfate can improve the symptoms of fatigue in pSD patients (Ng and Bowman, 2010), which may be related to its ability to control inflammation. In RCTs, missing some appointments or withdrawal from the trial often results in missing data and fewer available values. An MMRM is commonly used to evaluate repeated measures of clinical indicators, and unlike repeated measures analysis of variance (RMANOVA), it can make full use of all available observations and adapts well to missing data values. In this trial, there were a total of five results for each clinical symptom score at the different time points, but some patients had missing data because they did not return to the hospital at week 12. MMRM analysis demonstrated that except for the fatigue VAS score, significant group-by-time point interaction effects were observed for all symptom scale scores at 0 w-12 w time point, indicating that HRD requires a certain amount of time to improve patients’ symptoms, but at least 12 weeks.

In 2022, the New Clinical Endpoints in primary Sjögren’s syndrome: an interventional trial based on stratifying patients (NECESSITY) consortium developed the STAR composite response index as a tool for evaluating the results of RCTs on pSD (Seror et al., 2022). There was no significant difference in the STAR index between the two groups in our study, but the ClinESSDAI decreased significantly in the HRD group, and the results were better than those in the control group. This suggests that the evaluation effect of the ClinESSDAI on RCTs may be better than that of the ESSDAI to a certain extent, as concluded by the expert consensus in the development of the STAR tool (Seror et al., 2022). Previous studies have shown that long-term administration of hydroxychloroquine sulfate in pSD patients can effectively reduce the level of serum immunoglobulin (Kruize et al., 1993), but in our study, both groups achieved only limited serum indicator control, possibly due to the short course of treatment. The results of MMRM analysis revealed that the clinical symptom scores in the HRD group improved gradually with time over the treatment period and that group-by-time interaction effects were present from 0 w-12 w time point, while all the indicators recovered during the follow-up period, suggesting that there may be long-term deficiencies in the 12th week HRD treatment regimen employed in this study.

RNA methylation is an important form of gene posttranscriptional modification (Shi et al., 2019) and most commonly takes the form of m6A and m5C modifications (Chen et al., 2021; Courtney et al., 2019). Dynamic changes in m6A and m5C are widely involved in various immune reactions in SjD and play important roles in the activation and infiltration of immune cells (Gao et al., 2020; Zhou et al., 2021). In this study, we identified abnormal RNA modifications in pSD patients, mainly manifesting as a decrease in the m6A level and an increase in the m5C level; moreover, HRD reversed these changes to a certain extent. Few studies have investigated overall changes in RNA modifications, and most of them have focused on changes in regulatory factors. Some studies have suggested that certain regulators of m6A, such as METTL3 and ALKBH5, are related to disease activity and the level of inflammation in pSD patient (Cheng et al., 2022; Xiao et al., 2022). Moreover, there are few studies on regulators of m5C in pSD; one bioinformatics study has suggested that TET2 may be involved (Liu Y et al., 2023). We further analysed the expression levels of these regulators in the PBMCs of some participants by a microarray. Unlike previous studies, we found that HNRNPA2B1 may play a key role in the abnormal methylation identified in pSD. Previous studies have shown that HNRNPA2B1, as a nuclear DNA sensor, plays a role in recognizing viruses, activating the IFN pathway and triggering the immune response during the immune process (Wang et al., 2019), which is consistent with the pathogenesis of pSD (Otsuka et al., 2022). Moreover, in inflammatory bowel disease, HNRNPA2B1 has been confirmed to affect the infiltration of inflammatory factors by regulating the balance of T cells, thereby causing damage to epithelial cells (Meng et al., 2023). The findings of both that report and the present study suggest that RNA methylation can be used as an important epigenetic mechanism in pSD and that its regulators can be used as potential targets for the treatment of pSD. RF, an autoantibody, is involved in various autoimmune diseases. Studies have shown that in pSD, RF often causes elevated inflammatory responses and stronger immune activity during the course of the disease, which is an important factor affecting patient outcomes (Maślińska et al., 2021). HNRNPA2B1 has been shown to affect the level of inflammation in the body. In inflammatory bowel disease, this regulatory factor affects macrophage polarization, leading to disease progression (Meng et al., 2023). In this study, HNRNPA2B1 expression levels in pSD patients were positively correlated with serum RF levels, suggesting that RNA methylation-related regulatory factors may affect immune regulation processes through multiple pathways and can serve as new targets for disease diagnosis and treatment.

In conclusion, HRD combined with hydroxychloroquine sulfate can relieve the clinical symptoms, disease activity and exocrine gland function of pSD patients. Moreover, epigenetically, HRD can alter the levels of m6A and m5C in pSD patients, potentially by alterations to the expression of HNRNPA2B1 as a key regulator. The number of patients and test samples included in this study is limited, and this study excluded Sjögren associated to other autoimmune diseases, so the generalizability of the research conclusions is limited. In the future, we look forward to a large-sample, multicentre RCT to provide more evidence for the clinical application of the HRD. Moreover, only two forms of RNA methylation were detected, and future studies should consider investigating other forms of this modification.

## Data Availability

The raw data supporting the conclusions of this article will be made available by the authors, without undue reservation.

## Abbreviations

ACR/EULAR, American College of Rheumatology/European League Against Rheumatism; ANA, Antinuclear antibodies; ClinESSDAI, Clinical European League Against Rheumatism Sjögren’s Syndrome Disease Activity Index; CRP, C-reactive protein; DEG, Differential expression gene; ESR, Erythrocyte sedimentation rate; ESSDAI, European League Against Rheumatism Sjögren’s Syndrome Disease Activity Index; ESSPRI, European League Against Rheumatism Sjögren’s Syndrome Patient-Reported Index; HRD, Huashi Runzao decoction; IgA, Immunoglobulin A; IgG, Immunoglobulin G; IgM, Immunoglobulin M; LC-MS, Liquid chromatography–mass spectrometry; m5C, 5-methylcytosine; m6A, N6-methyladenosine; MMRM, Mixed-effect model for repeated measures; NECESSITY, the New Clinical Endpoints in primary Sjögren’s syndrome: an interventional trial based on stratifying patients; PBMC, Peripheral blood mononuclear cell; pSD, Primary Sjögren disease; RCT, Randomized controlled trial; RF, Rheumatoid factor; SF-36, Short form-36 health survey; SjD, Sjögren disease; SSA, Sjögren’s -syndrome-related antigen A; SSB, Sjögren’s -syndrome-related antigen B; STAR, Sjögren’s tool for assessing response; VAS, Visual analogue scale.
