# Supplementary figures and images for: Huashi Runzao decoction for primary Sjögren disease: a double-blind, randomized controlled trial combined with m6A and m5C RNA modification analysis
Source: Front Pharmacol. 2025 Oct 31;16:1618649. doi: 10.3389/fphar.2025.1618649 (PMC12615371; doi:10.3389/fphar.2025.1618649)

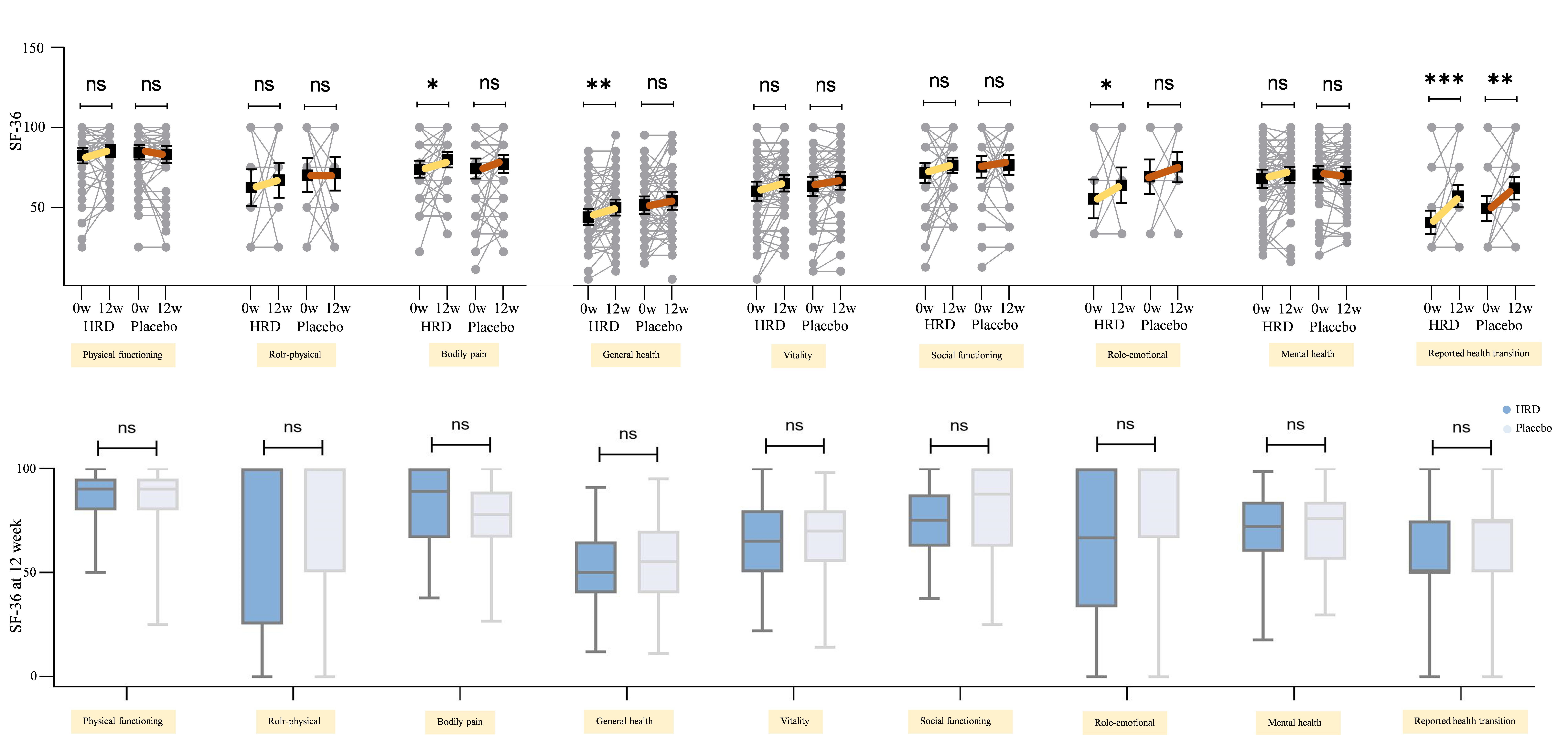

Supplement: Supplementary file 1 [file Image2.tif]

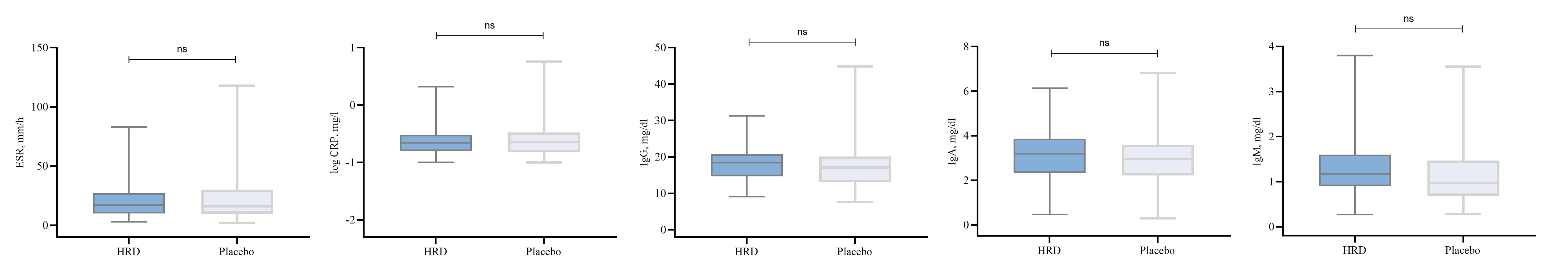

Supplement: Supplementary file 2 [file Image1.tif]
